# On the dosimetric impact of inhomogeneity management in the Acuros XB algorithm for breast treatment

**DOI:** 10.1186/1748-717X-6-103

**Published:** 2011-08-26

**Authors:** Antonella Fogliata, Giorgia Nicolini, Alessandro Clivio, Eugenio Vanetti, Luca Cozzi

**Affiliations:** 1 Oncology Institute of Southern Switzerland, Medical Physics Unit, Bellinzona, Switzerland

**Keywords:** Acuros, AAA, breast, inhomogeneity correction, tissue density

## Abstract

**Background:**

A new algorithm for photon dose calculation, Acuros XB, has been recently introduced in the Eclipse, Varian treatment planning system, allowing, similarly to the classic Monte Carlo methods, for accurate modelling of dose deposition in media. Aim of the present study was the assessment of its behaviour in clinical cases.

**Methods:**

Datasets from ten breast patients scanned under different breathing conditions (free breathing and deep inspiration) were used to calculate dose plans using the simple two tangential field setting, with Acuros XB (in its versions 10 and 11) and the Anisotropic Analytical Algorithm (AAA) for a 6MV beam. Acuros XB calculations were performed as dose-to-medium distributions. This feature was investigated to appraise the capability of the algorithm to distinguish between different elemental compositions in the human body: lobular vs. adipose tissue in the breast, lower (deep inspiration condition) vs. higher (free breathing condition) densities in the lung.

**Results:**

The analysis of the two breast structures presenting densities compatible with muscle and with adipose tissue showed an average difference in dose calculation between Acuros XB and AAA of 1.6%, with AAA predicting higher dose than Acuros XB, for the muscle tissue (the lobular breast); while the difference for adipose tissue was negligible. From histograms of the dose difference plans between AAA and Acuros XB (version 10), the dose of the lung portion inside the tangential fields presented an average difference of 0.5% in the free breathing conditions, increasing to 1.5% for the deep inspiration cases, with AAA predicting higher doses than Acuros XB. In lung tissue significant differences are found also between Acuros XB version 10 and 11 for lower density lung.

**Conclusions:**

Acuros XB, differently from AAA, is capable to distinguish between the different elemental compositions of the body, and suggests the possibility to further improve the accuracy of the dose plans computed for actual treatment of patients.

## Background

Radiotherapy in the management of early stage breast cancer after surgery contributes to a fundamental reduction of the risk of local relapse. From the dosimetric point of view, the task of generating treatment plans of high quality is challenged by the complex anatomy of the thoracic district due to the neighbourhood of tissues of highly different density, composition and homogeneity, especially the lungs with a density much lower than the surrounding soft tissues. Taking benefit from geometrical features, it has been proven [1,2] that for breast treatment, the usage of specific respiratory gating phases, namely deep inspiration, might be dosimetrically beneficial. This because of the increased separation between the heart and the chest wall which is maximized in that respiratory phase [1]. A second benefit derived from the remarkable reduction of the density of the lung parenchyma, a fact that correlates to additional dose reduction [3,4]. To assess the benefit from the second feature, it is necessary to perform dose calculations with accurate algorithms, capable to properly model radiation transport in all media. It is nevertheless a fact that most of the photon dose calculation engines have a more or less limited accuracy in predicting dose in low density media than in higher density tissues [5-7], especially those algorithms that use heavy approximations in modelling the lateral electron transport (e.g. convolution/superposition methods).

To improve dose calculation in heterogeneous tissues, some algorithms implement the possibility to account for the specific elemental composition of the human body. This is typically realised by associating the Hounsfield Units from the CT scans to a mass density and material derived from customised and simplified conversion tables where, for predefined density ranges, specific elemental composition are assigned. In general the composition is taken from data repositories based on general consensus as, for example, the ICRP Report 23 [8]. Algorithms capable to incorporate tissue composition in the dose calculation mechanisms have an increased accuracy in determining the dose to each specific organ [9,10]. In the case of breast treatments, beside the need of properly modelling the lung tissue (with complex composition and very low density at the same time when deep inspiration breathing is considered), also inhomogeneities in the region of the target volume should be carefully modelled since the mammary gland has a quite complex structure as well.

Aim of the present study is the assessment of the dosimetric impact of a new dose calculation algorithm on datasets from a cohort of real patients where a variety of different breast tissue densities and lung air filling are in place.

The new algorithm under investigation is the Acuros^® ^XB Advanced Dose Calculation (Acuros XB) as it is implemented in the Eclipse treatment planning system (Varian Medical Systems, Palo Alto, USA). This algorithm belongs to the class of the Linear Boltzmann Transport Equation (LBTE) solvers, allowing, similarly to the classic Monte Carlo methods, for accurate modelling of dose deposition in heterogeneous media [11-13]. In the study, calculations performed with Acuros XB are evaluated against the well known and validated Anisotropic Analytical Algorithm (AAA) similarly implemented in the Eclipse planning system [14-16].

## Methods

### A. Patient selection and planning techniques

CT data from 10 patients presenting left side breast carcinoma were selected for the study. For all patients two scanning acquisition sets were available: the first leaving the patient to normally breath (free breathing, FB), the second obtained by scanning patients under maximum inhale and breath hold condition during the whole CT acquisition (deep inspiration breath hold, DIBH). Gating and breath tracking during scanning were determined by means of the Respiratory Gating RPM system (Varian Medical System, Palo Alto, CA); adjacent slices with 5 mm thickness were acquired on a 16 slices scanner with an acquisition time of the entire thorax region of about 8 seconds.

Both CT datasets were contoured, for each patient, with planning target volume (PTV), left and right lungs, heart, and contra lateral breast. PTV on the two CTs were carefully drawn considering anatomical landmarks; each pair of PTV volumes differed less than 5%.

Dose plans were computed for conventional conformal techniques based on two tangential fields (average field size of 18.9 ± 0.8 cm in the longitudinal direction and 11.1 ± 1.6 cm in the transversal direction) using 6MV beams from a Varian Clinac equipped with a standard 80-leaf MLC; dynamic wedges (EDW) were used whenever needed. As a common strategy, a first plan, forward optimized with trial and error procedure, was obtained for the DIBH cases, and a second plan with the same beam characteristics of gantry angles and wedges was computed for each corresponding FB CT (adjusting MLC shapes and beam weights if needed).

Dose prescription was set to 50 Gy at 2 Gy/fraction, to the mean target dose.

### B. Dose calculation algorithms

All plans (in number of two plans per each patient, for FB and DIBH CT acquisitions respectively) were computed with the following dose calculation algorithms, all implemented in the platform version 10 of the Eclipse treatment planning system (Varian Medical System):

- Acuros XB: Acuros^® ^XB Advanced Dose Calculation, version 10.0.28, the first version released for clinical use.

- Acuros XB: Acuros^® ^XB Advanced Dose Calculation, version 11.0.02, a pre-clinical engineering release.

- AAA: Anisotropic Analytical Algorithm, clinical version 10.0.28.

Calculation grid was set to 2.5 mm in all cases. All AAA and Acuros XB plans were calculated for the same number of MU.

Acuros XB algorithm solves numerically the Linear Boltzmann Transport Equation (LBTE) which describes the macroscopic behaviour of radiation particles as they travel through and interact with matter. It allows, similarly to the classic Monte Carlo methods, for accurate modelling of dose deposition in heterogeneous media. The original Acuros algorithm for external beams is published by Vassiliev *et al *[13]. Its implementation in Eclipse is briefly described in Fogliata *et al *[17].

Acuros XB implementation in Eclipse consisted on two parts: the photon beam source model and the radiation transport model. The first one was realised with the same multiple source model already implemented in Eclipse for AAA and was described in detail in Tillikainen *et al *[18]. Concerning the radiation transport model, Acuros XB can calculate the dose to water or dose to medium, accounting for the elemental composition of specific anatomical regions as derived by the CT dataset. Tissue segmentation is automatically performed based on density ranges derived from the HU values read in the CT dataset of the patients. Table 1 reports the correspondence matrix for the segmentation from density to human tissues for the two versions of Acuros XB used in the study. For each material, the specific chemical elemental composition is based on the ICRP Report 23 [8]. In addition, Acuros XB does not perform automatic material assignment to any voxel that has an HU value larger than the maximum HU value in the CT calibration curve, or that has a mass density higher than 3.0 g/cm^3^. If CT dataset contains voxels that exceed these limits, the user must create a structure and manually assign the material and mass density.

**Table 1:** Material mass densities

	Acuros XB vers. 10		Acuros XB vers. 11	
**Material**	**Lowest Density**	**Highest Density**	**Lowest Density**	**Highest Density**

Air	-	-	0.000	0.0204
Lung	0.000	0.590	0.011	0.6242
Adipose Tissue	0.590	0.985	0.5539	1.001
Muscle, Skeletal	0.985	1.075	0.9693	1.0931
Cartilage	1.075	1.475	1.0556	1.600
Bone	1.475	3.000	1.100	3.000

Material mass densities in g/cm^3 ^for automatic material conversion, as implemented in the two Acuros XB versions.

One of the main differences between the two analysed Acuros XB versions (10 and 11) is given by the different strategy in the density-to-media assignment, as shown in Table 1. With respect to version 10, version 11 includes some refinements. Firstly, automatic assignment of the Air material to very low density regions inside body was implemented. Secondly, the density range per each material was slightly extended with an overlap of densities between adjacent materials. In the overlapping range, the elemental composition is considered as a proportional mixture of the previous and next material. Note the large overlap between cartilage and bone; for these two tissues, the difference in calcium content plays a fundamental role in the dose calculation phase (to medium and/or water).

First validations of Acuros XB implementation in Eclipse can be found in Fogliata *et al *[17] and in Bush *et al *[19].

AAA is an analytical photon dose calculation algorithm based on a pencil-beam convolution/superposition technique; in the lateral scaling of the medium it applies six independent exponential absorption functions to account for the lateral transport of energy with varying densities. The algorithm was originally founded on the works of Ulmer *et al *[14,15,20], and Tillikainen *et al *[16,18]. AAA was extensively validated against phantom measured data [21-23], or mainly to focus on heterogeneity issues [6,24]. Readers should refer to Tillikainen *et al *for detailed description [16].

### C. Breast and lung densities

Since Acuros XB implemented tissue composition modelling, some detailed features of the two main tissues involved in the clinical case under investigation are here specified, reporting dose to medium.

#### Lung tissue

lung densities were compared for the two breathing acquisitions, and comprehensive data can be found in Fogliata *et al *[3]. For the cohort of patients in the present study, the ratio between mean lung volumes in DIBH and FB was 1.76 ± 0.20, and the average values of HU were -826 ± 17 and -723 ± 35 for DIBH and FB modes (p < < 0.0001 with a paired t-Student test), respectively, corresponding to mean lung densities of 0.15 ± 0.02 and 0.26 ± 0.04 g/cm^3^.

#### Breast tissue

anatomically, the mammary gland consists of various compartments, separated by adipose tissue; each compartment consists of smaller lobules composed of connective tissue. From ICRP-89 [25] the glandular fraction is assumed to be about the 40% of the entire breast. In female, the breast composition (including glandular fraction and adipose) presents lower carbon and higher oxygen fractions than fat [25]. This different elemental composition of glandular fraction and fat is reflected in the muscle and adipose human materials [8,26].

### D. Data evaluation

Analysis of dose calculations in lung tissue was performed through dose plan differences between AAA and the two Acuros XB versions, as well as difference between the two Acuros XB versions, for both DIBH and FB. In this context two lung sub-structures were considered: Lung_IN and Lung_OUT (being Lung the entire lung structure, that is also the union of Lung_IN and Lung_OUT). Lung_IN is the lung portion falling geometrically inside the projection of the edges of the two tangential radiation fields. Lung_OUT is its complement, i.e. the portion of lung outside the field edges. It is thus possible to analyse the behaviour of the algorithms when primary radiation transport dominates or where mostly scattering shall be the dominant component to dose deposition. Numerically, mean and standard deviations were recorded for Lung_IN, Lung_OUT and Lung from dose difference plans for each patient in DIBH and FB and then averaged over the whole patient cohort. To better visualize the global pattern of differences, the average differential histograms relative to dose difference plans for each structure were plotted in the various conditions.

For target breast soft tissue, the analysis was conducted aiming to appraise the difference in dose calculations in the two breast components, the one composed by lobular breast tissue (segmented as Muscle tissue for calculations), and the one composed by fat (Adipose tissue for calculations). To achieve this aim, two PTV sub- structures were defined: PTV_musc and PTV_adip, the first having density higher than 0.985 g/cm^3^, the second lower than this value.

Numerical analysis of Dose Volume Histograms DVH was performed for all difference plans couples: AAA - Acuros XB version 10, AAA - Acuros XB version 11, and Acuros XB version 10 - Acuros XB version 11. The last couple aimed to demonstrate the impact of a more sophisticated management of density to tissue conversion.

To assess how different dose calculations for specific lung density related to different air filling, or different soft tissue composition is detectable in terms of clinical appraisal using a different algorithm, comparisons were performed through mean dose and V_x _values from DVH, with × = 5, 10, 20, 40, 45 Gy. Some data comparison between DIBH and FB for the three lung structures, and between PTV_musc and PTV_adip for PTV volume were reported.

In the present paper the comparison between the two algorithms would evidentiate both the differences arising by the algorithm per se, and the usage in clinical cases of the dose to medium (with the consideration of the elemental composition as with Acuros XB), or dose to water (indeed rescaled to water as with AAA). A fair comparison between the two algorithms in the same frame of dose calculation rescaled to water has been published in Fogliata *et al *[27].

## Results

**Figure 1:**
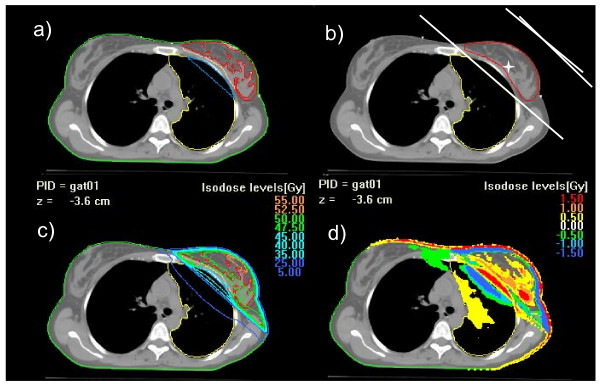
**Axial view of an example case**: a) Lung_IN (light blue) and Lung_OUT (yellow) contours for lung; PTV_musc (pink) and PTV_adip (red) contours for target breast; b) treatment technique of two tangential fields; c) dose distribution for Acuros XB version 10 calculations; d) dose distribution for the plan difference AAA-Acuros10.

Figure 1 shows an example of an axial view of a patient with beam arrangement and contoured sub-structures is presented, together with dose difference patterns.

### A. Lung tissue

**Figure 2:**
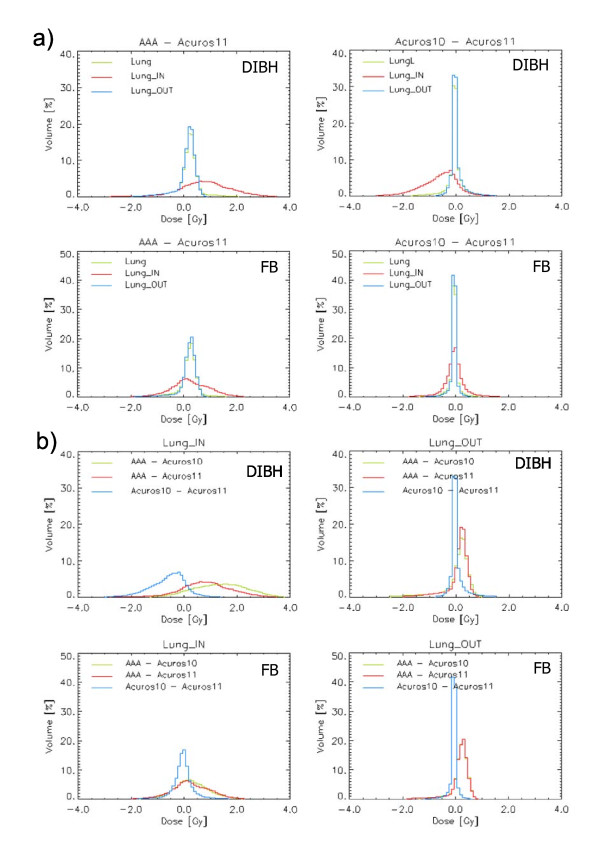
**Differential lung Dose-Volume Histograms of the difference plan**: a) for FB (left) and DIBH (right), plots for the entire lung and the two lung sub-structures; first row: AAA-Acuros version 10, second row: Acuros version 10-Acuros version 11. b) for FB (left) and DIBH (right), plots for all dose difference plans; first row: Lung_IN structure, second row: Lung_OUT structure.

Results of the dose calculations for different lung density in the two different lung regions are summarised in Table 2 and in Figure 2. Table 2 reports, for lung tissues, the values of the mean and the standard deviation (average ± SD and range over all the ten patients) of the histograms of the dose difference plans between two calculations algorithms, in particular AAA-Acuros11 and Acuros10-Acuros11. Lung_IN and Lung_OUT structures were considered separately for the two air filling conditions of the lung, i.e. FB and DIBH, not having the possibility to register with a deformable algorithm structures and doses. Figure 2 reports the histograms averaged over all the patients, for the two lung portions as well as for the entire lung, for all the difference plans. Data shows a significant dose difference inside the field (Lung_IN) between AAA and Acuros XB in the two air filling, being the average variation of 0.5% in the FB case (p < 10^-4 ^with a t-Student test), value that increases to 1.5% in the DIBH case (p < 10^-4 ^with a t-Student test). AAA calculations predicts higher dose than Acuros XB. Looking at the two Acuros XB versions, negligible difference of 0.2% is shown in the FB case, while an average of 1.3% (p < 10^-4 ^with a t-Student test) is obtained for the lower density case of lung, resulting in higher dose computed by version 11. The difference arises from the inclusion, in the list of materials, of the air for very low density pixels (being pure air up to 0.011 g/cm^3^, and a mixture of air and lung tissues from 0.011 to 0.0204 g/cm^3^), together with a more accurate calculation for very low density lung, implemented in version 11 of Acuros XB. On the contrary, the difference in dose calculations outside the field (due to scattering) is negligible among all algorithms and lung densities.

**Table 2:** Mean structure values of difference plans

		AAA-Acuros11		Acuros10-Acuros11	
		
		Mean ± SD	Range	Mean ± SD	Range
Lung	Mean %	0.3 ± 0.2	[0.0, 0.7]	-0.1 ± 0.2	[-0.4, 0.3]
DIBH	Std.Dev. %	1.3 ± 0.2	[1.0, 1.5]	0.8 ± 0.2	[0.5, 1.1]

Lung_IN	Mean %	1.5 ± 1.5	[-1.3, 3.3]	-1.3 ± 1.1	[-3.5, 0.4]
DIBH	Std.Dev. %	1.8 ± 0.4	[1.4, 2.8]	1.2 ± 0.3	[0.8, 1.7]

Lung_OUT	Mean %	0.1 ± 0.1	[-0.1, 0.4]	0.0 ± 0.2	[-0.2, 0.3]
DIBH	Std.Dev. %	1.0 ± 0.2	[0.8, 1.5]	0.6 ± 0.2	[0.4, 0.9]

Lung	Mean %	0.3 ± 0.2	[-0.1, 0.7]	-0.2 ± 0.2	[-0.6, 0.2]
FB	Std.Dev. %	1.0 ± 0.2	[0.8, 1.4]	0.5 ± 0.2	[0.3, 0.9]

Lung_IN	Mean %	0.5 ± 0.6	[-0.5, 1.5]	-0.1 ± 0.6	[-0.9, 0.9]
FB	Std.Dev. %	1.5 ± 0.1	[1.3, 1.6]	0.8 ± 0.2	[0.4, 1.1]

Lung_OUT	Mean %	0.3 ± 0.2	[0.0, 0.5]	-0.2 ± 0.2	[-0.5, 0.1]
FB	Std.Dev. %	0.9 ± 0.3	[0.6, 1.4]	0.5 ± 0.2	[0.2, 0.8]

PTV	Mean %	0.3 ± 0.7	[-0.9, 1.4]	0.1 ± 0.2	[-0.1, 0.6]
	Std.Dev. %	2.7 ± 1.0	[1.6, 4.6]	1.5 ± 0.8	[0.8, 3.4]

PTV_muscle	Mean %	1.6 ± 0.3	[1.1, 2.1]	-0.1 ± 0.1	[-0.4, 0.1]
	Std.Dev. %	1.4 ± 0.4	[1.1, 2.4]	1.0 ± 0.4	[0.5, 2.0]

PTV_adipose	Mean %	-0.2 ± 1.2	[-2.8, 1.3]	0.2 ± 0.3	[-0.1, 0.8]
	Std.Dev. %	2.9 ± 1.2	[1.6, 5.2]	1.6 ± 0.9	[0.8, 3.7]

Mean and Standard Deviations parameters (in percentage) of the differential DVH for AAA-Acuros11 and Acuros10-Acuros11 difference plans. Both parameters are recorded as Mean ± SD and range for lung, PTV and their sub-structures. For lung contours, both DIBH and FB modes are reported, while for PTV contours only DIBH is shown.

In Figure 2 the distribution of the dose differences is shown also for the entire lung tissue. Due to the rather small portion of lung volume included in the fields (Lung_IN is 11 ± 3% for DIBH and 15 ± 4% for FB of the whole lung volume averaged over the ten analysed patients), the systematic difference of the dose calculations would have been hidden if the entire volume was used. From Figure 2 and Table 2 it is visible also the rather large spread (standard deviation of the histograms) of the difference between AAA and Acuros XB in Lung_IN. This spread decreases between the two Acuros XB versions, but only in the FB cases.

### B. Soft tissue

**Figure 3:**
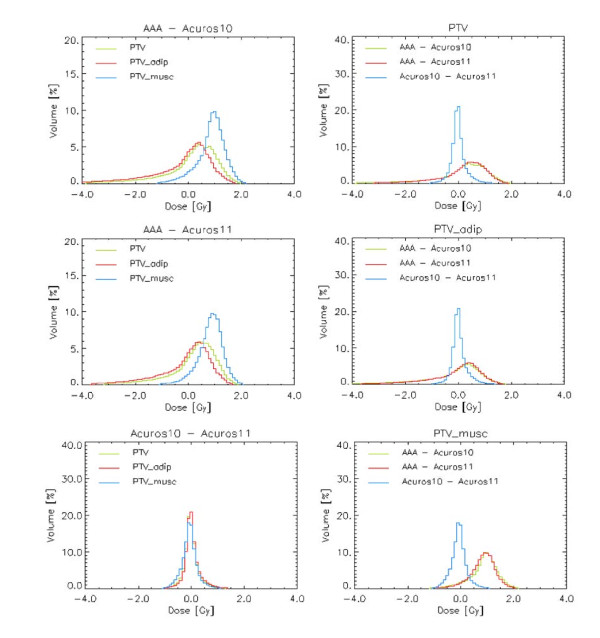
**Differential PTV Dose-Volume Histograms of the difference plan (DIBH mode only); first column: for each dose difference plan the entire PTV and the two PTV sub-structures are plotted; second column for each PTV structure the three dose difference plans are plotted**.

The results of the analysis of the target volume and its stratification in the two sub-structures PTV_musc and PTV_adip are reported in Table 2 and Figure 3, from dose difference plan calculations. The PTV analysis is reported only for DIBH cases. The FB cases were analysed as well, and the results were similar. From Table 2 the difference in dose calculation between AAA and Acuros XB in muscle tissue is in average 1.6%, with AAA predicting higher dose than Acuros XB. The same metric for adipose tissue gives negligible differences (0.2%). Between the two Acuros XB versions, almost no difference is found (being in average within 0.2% for the two tissue materials). This last absence of difference was expected, because the mean densities of PTV_musc and PTV_adip, of 1.013 and 0.954 g/cm^3 ^respectively, lie well within the range of the corresponding material, and almost no mixed tissue is considered in version 11.

From the histograms plotted in Figure 3, it is clear that the systematic difference in dose calculation in the muscle tissue of the breast would have been hidden if only the PTV was analysed, as the adipose tissue composing the breast is in average, over the analysed patients, 74% (ranging from 42 to 89%) of the whole target.

### C. Clinical appraisal from global DVH

**Figure 4:**
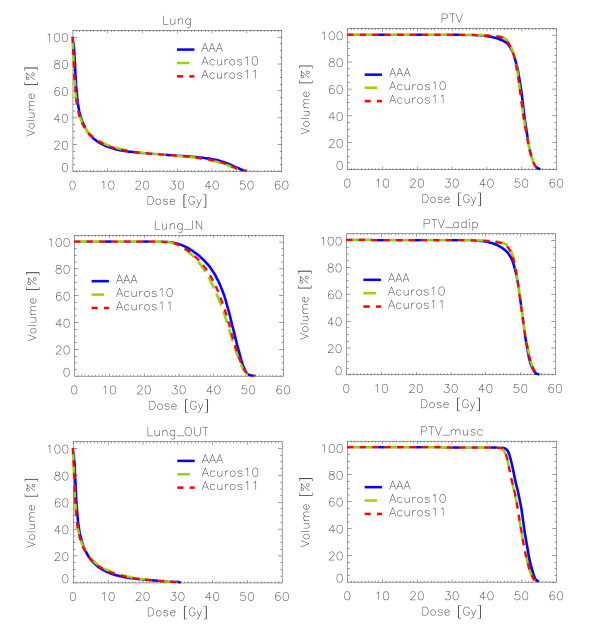
**Cumulative average DVH of the three lung and PTV structures for the three analysed dose calculation algorithms, for DIBH mode cases**.

Results for the statistical parameters from DVH are summarised in Table 3 (as mean values and standard deviations over all patients) for PTV and its two components, PTV_musc and PTV_adip, and for Lung and its two components, Lung_IN and Lung_OUT. Plots of the average cumulative histograms for DIBH cases are presented in Figure 4. If plans are compared only for the entire lung and PTV, as generally done in clinical practice, AAA and Acuros XB would show minor differences. When the two subcomponents of the two main structures are, analysed, the differences become relevant also in terms of cumulative DVH. For example the shift of DVH toward high doses is clear for PTV_musc and Lung_IN. From statistics differences are visible for Lung_IN calculations, where also the difference between the two Acuros XB versions is evident for DIBH cases for the V_40Gy _parameter. Regarding PTV, a significant difference between AAA and Acuros XB calculations is visible only in the two PTV sub-structures, where V95% shows, for Acuros XB calculations, higher values in the adipose tissue, and lower values in the muscle tissue.

**Table 3:** DVH statistics

	DIBH			FB		
	
	AAA	Acuros10	Acuros11	AAA	Acuros10	Acuros11
***Lung***						
Mean [Gy]	7.7 ± 1.5	7.5 ± 1.5	7.5 ± 1.5	9.2 ± 1.9	9.0 ± 1.9	9.0 ± 1.9
V10Gy [%]	18.2 ± 3.6	19.5 ± 3.7	19.0 ± 3.6	21.4 ± 4.6	21.9 ± 4.6	21.9 ± 4.6
V20Gy [%]	13.4 ± 3.4	13.7 ± 3.4	13.6 ± 3.4	16.9 ± 4.3	17.0 ± 4.3	17.0 ± 4.3
V40Gy [%]	8.9 ± 2.9	7.7 ± 2.9	8.2 ± 2.9	11.6 ± 3.5	11.2 ± 3.5	11.1 ± 3.5

***Lung_IN***						
Mean [Gy]	43.0 ± 1.4	41.5 ± 1.7	42.0 ± 1.5	43.4 ± 0.9	43.1 ± 1.0	43.0 ± 1.0
V40Gy [%]	77.2 ± 5.8	66.3 ± 9.3	70.4 ± 7.6	78.6 ± 3.9	75.7 ± 4.9	75.2 ± 4.9

***Lung_OUT***						
Mean [Gy]	3.1 ± 0.4	3.1 ± 0.4	3.0 ± 0.4	3.3 ± 0.4	3.0 ± 0.4	3.1 ± 0.4
V10Gy [%]	7.6 ± 1.2	9.0 ± 1.4	8.5 ± 1.3	7.9 ± 1.4	8.4 ± 1.5	8.4 ± 1.5
V20Gy [%]	2.2 ± 0.5	2.5 ± 0.5	2.4 ± 0.5	2.6 ± 0.8	2.7 ± 0.8	2.7 ± 0.8

***PTV***						
Mean [Gy]	50.0 ± 0.0	49.9 ± 0.3	49.9 ± 0.3	50.0 ± 0.0	49.9 ± 0.3	49.9 ± 0.3
St. Dev. [Gy]	2.8 ± 0.6	2.4 ± 0.5	2.5 ± 0.6	2.4 ± 0.3	2.1 ± 0.2	2.2 ± 0.2
V90% [%]	95.2 ± 2.3	97.8 ± 1.6	97.3 ± 1.8	96.0 ± 1.9	98.7 ± 0.9	98.3 ± 1.0
V95% [%]	87.4 ± 3.0	88.2 ± 2.9	87.5 ± 2.8	87.8 ± 3.2	87.9 ± 2.9	87.2 ± 2.8
V107% [%]	5.0 ± 2.1	5.2 ± 2.1	4.7 ± 2.0	3.2 ± 1.3	3.8 ± 2.1	3.4 ± 2.0

***PTV_adip***						
Mean [Gy]	49.8 ± 0.4	50.2 ± 0.3	50.1 ± 0.3	49.8 ± 0.5	50.1 ± 0.3	50.0 ± 0.3
St. Dev. [Gy]	2.9 ± 0.7	2.5 ± 0.6	2.6 ± 0.6	2.6 ± 0.5	2.1 ± 0.3	2.2 ± 0.4
V90% [%]	93.7 ± 3.6	97.9 ± 1.4	97.2 ± 1.7	94.3 ± 4.3	98.5 ± 1.1	98.1 ± 1.5
V95% [%]	86.1 ± 6.6	91.0 ± 1.0	90.0 ± 1.2	86.0 ± 7.4	90.0 ± 2.3	89.4 ± 2.6
V107% [%]	4.8 ± 2.3	6.2 ± 2.6	5.6 ± 2.4	3.4 ± 1.9	5.0 ± 3.5	4.4 ± 3.1

***PTV_musc***						
Mean [Gy]	50.0 ± 0.8	49.1 ± 0.9	49.1 ± 0.9	50.0 ± 0.8	49.0 ± 0.8	49.1 ± 0.8
St. Dev. [Gy]	2.1 ± 0.4	2.2 ± 0.4	2.2 ± 0.4	1.9 ± 0.4	2.1 ± 0.4	2.0 ± 0.4
V90% [%]	99.4 ± 0.8	97.8 ± 2.4	97.8 ± 2.3	99.8 ± 0.2	98.6 ± 2.1	98.7 ± 2.0
V95% [%]	85.3 ± 12.6	72.6 ± 19.6	72.9 ± 19.8	86.4 ± 10.2	70.7 ± 19.1	71.1 ± 19.2
V107% [%]	5.5 ± 2.6	2.4 ± 1.5	2.2 ± 1.6	3.6 ± 3.4	1.5 ± 1.9	1.3 ± 1.8

Statistics for lung and PTV structures in DIBH and FB modes, from all three analysed algorithms and versions.

## Discussion

The present study aimed to investigate the performance, for a given clinical model, of the new Acuros XB algorithm for photon dose calculations recently implemented in the Eclipse planning system, in comparison with the commonly used AAA algorithm. Focus was put on two main general criticalities. The first is the behaviour of the algorithm in lungs when different air filling and density has to be considered due to different respiratory conditions, i.e. FB and DIBH. The second is the capability of the dose calculation engine to distinguish between different types of soft tissues characterised by significantly different chemical composition but anatomically strongly interlaced: the lobular gland (muscle) and adipose tissue, having different elemental composition in terms of carbon and oxygen proportions.

The two photon dose calculation algorithms here analysed, implement totally different approaches, and, for the subject of the study, the main point is focussed to the capability, for Acuros XB, to manage elemental compositions of some predefined human tissues, and therefore to calculate the dose to proper medium. Those characteristics are not available in AAA, where the calculation accounts only for the different densities of the materials, but the dose is computed as dose to density rescaled water. From Acuros XB validation in water and in heterogeneous media [17,19,27], benchmarked respectively against measurements and Monte Carlo calculations, it has been shown that differences between AAA and Acuros XB calculations can be interpreted as an improvement in accuracy when using the newer algorithm.

Considering the lung dose calculations, the difference between algorithms was found in the region within the two tangential fields. The greatest differences, as expected, were found in the DIBH cases, presenting the lowest lung densities (0.15 g/cm^3 ^with respect to 0.26 g/cm^3 ^in the same FB cases). In this region the AAA dose overestimation is in average of 1.5% (with a maximum value of 3.3% in the patient cohort). Presenting, on the contrary, very negligible differences in the region out of the field between the two algorithms, the offset here measured is generally not visible in the common practice of inspecting DVH. The same effect is the difference of dose calculated in the muscle tissue of the breast, and again not visible in common DVH analysis being the muscle tissue only one fourth of the entire target breast volume. In this last case the 1.6% average overestimation (maximum value 2.1%) of AAA calculation should be read with a different approach: the mean dose to the adipose tissue of the entire breast is very near to the prescription dose, while the mean dose to the muscle tissue, that is indeed the true mammary tissue, is lower than prescription of almost 1 Gy for a common 50 Gy treatment. This means that, in the conformal treatment with two tangential fields where no modulation is foreseen, and prescribing the treatment to the mean target dose, a systematic underdosage of about 1 Gy of the muscle-like tissue of the breast could be delivered due to the difference in dose distribution (not necessarily in dose calculation) in the two different breast components. The specific amount of the underdosage and its distribution within the breast is clearly depending on the patient anatomy. To consider, on the other side, is that the implemented table relating HU, mass density and finally elemental composition of the patient body, is a strong approximation of what could be the real composition of the patient. In principle this could result in attributing the relative composition of components of an organ (e.g., oxygen or carbon that presents rather different stopping power) that diverges from the actual component, leading consequently to a calculated dose that diverges from the actual dose absorbed by the real tissue.

Summarising, even if with the commonly applied methods of plan comparison based on DVH analysis it is difficult to appraise significant differences between AAA and Acuros XB, those can be estimated by means of more detailed analysis of sub-structures of a same volume characterised by different compositions or dose intensity delivery. Once defined the possible source of differences between dose calculation algorithms, it is possible to appreciate the merit of using a highly sophisticated algorithms in the clinical practice. The availability of commercial algorithms capable to discriminate among different tissues and chemical composition (although using pre-defined and simplified segmentation methods) is of primary importance in order to better understand the dose that can actually be delivered to patients in anatomical sites known to be inaccurately managed by older algorithms.

## Conclusions

Improvements in dose calculations with the usage of sophisticated algorithms, and the possibility to account for proper elemental compositions of the various tissues of the human body allows a better knowledge of the actual dose distribution inside the patient, which in the future could better describe the clinical outcome in particular situations. In particular, the possibility to better compute the dose delivered to parts of specific organs, as in the breast example where the dose to the lobular of fat tissues is systematically different due to their elemental compositions, might make better understanding of toxicities or treatment outcome arising from such differences.

The availability of accurate algorithms give to the community an improvement in the consistency between actual and calculated treatment doses, a fact that can have a clinical impact on the consistency of data in clinical trials.

## Competing interests

Dr. L. Cozzi acts as Scientific Advisor to Varian Medical Systems and is Head of Research and Technological Development to Oncology Institute of Southern Switzerland, IOSI, Bellinzona.

No special competing interest exists for any other author.

## Authors' contributions

AF: study coordination, data analysis, manuscript preparation. GN, EV, AC: data analysis. LC: study coordination, manuscript preparation. All authors read and approved the final manuscript.
